# Fly Photoreceptors Demonstrate Energy-Information Trade-Offs in Neural Coding

**DOI:** 10.1371/journal.pbio.0050116

**Published:** 2007-03-20

**Authors:** Jeremy E Niven, John C Anderson, Simon B Laughlin

**Affiliations:** 1 Department of Zoology, University of Cambridge, Cambridge, United Kingdom; 2 Biology and Environmental Science, School of Life Sciences, University of Sussex, Brighton, United Kingdom; Harvard University, United States of America

## Abstract

Trade-offs between energy consumption and neuronal performance must shape the design and evolution of nervous systems, but we lack empirical data showing how neuronal energy costs vary according to performance. Using intracellular recordings from the intact retinas of four flies, Drosophila melanogaster, D. virilis, *Calliphora vicina,* and Sarcophaga carnaria, we measured the rates at which homologous R1–6 photoreceptors of these species transmit information from the same stimuli and estimated the energy they consumed. In all species, both information rate and energy consumption increase with light intensity. Energy consumption rises from a baseline, the energy required to maintain the dark resting potential. This substantial fixed cost, ∼20% of a photoreceptor's maximum consumption, causes the unit cost of information (ATP molecules hydrolysed per bit) to fall as information rate increases. The highest information rates, achieved at bright daylight levels, differed according to species, from ∼200 bits s^−1^ in D. melanogaster to ∼1,000 bits s^−1^ in S. carnaria. Comparing species, the fixed cost, the total cost of signalling, and the unit cost (cost per bit) all increase with a photoreceptor's highest information rate to make information more expensive in higher performance cells. This law of diminishing returns promotes the evolution of economical structures by severely penalising overcapacity. Similar relationships could influence the function and design of many neurons because they are subject to similar biophysical constraints on information throughput.

## Author Summary

Many animals show striking reductions or enlargements of sense organs or brain regions according to their lifestyle and habitat. For example, cave dwelling or subterranean animals often have reduced eyes and brain regions involved in visual processing. These differences suggest that although there are benefits to possessing a particular sense organ or brain region, there are also significant costs that shape the evolution of the nervous system, but little is known about this trade-off, particularly at the level of single neurons. We measured the trade-off between performance and energetic costs by recording electrical signals from single photoreceptors in different fly species. We discovered that photoreceptors in the blowfly transmit five times more information than the smaller photoreceptors of the diminutive fruit fly *Drosophila*. The blowfly pays a high price for better performance; its photoreceptor uses ten times more energy to code the same quantity of information. We conclude that, for basic biophysical reasons, neuronal energy consumption increases much more steeply than performance, and this intensifies the evolutionary pressure to reduce performance to the minimum required for adequate function. Thus the biophysical properties of sensory neurons help to explain why the sense organs and brains of different species vary in size and performance.

## Abstract 3

Evidence from single-neuron recordings supports the law of diminishing returns, i.e., high performance eyes in larger, faster flies have less efficient photoreceptors than those of their small, sluggish counterparts.

## Introduction

The balance between cost and benefit plays an important role in directing the evolution of biological systems [1,2]. Costs and benefits are many and various; for example, the elongated tail of the male long-tailed widow bird is very effective at attracting females, but it also makes the male more conspicuous to predators and greatly increases the energetic cost of flight [3,4]. Many of the costs that are incurred in the manufacture, maintenance, operation, and carriage of systems can be reduced to a common currency, the expenditure of metabolic energy, while the benefits can be measured in terms of a system's performance. A system's energy cost and its performance interact, within the context of the organism and its habitat, to determine fitness.

Relationships between cost and performance have undoubtedly shaped the evolution of nervous systems [5–7]. The enlargement of structures for particularly important and demanding behavioural tasks, such as the auditory system of a bat [8], and the reduction of redundant structures, such as the thalamo-cortical visual system of the subterranean mole rat *Spalax* [9], suggest that larger structures perform better and cost more. Economical wiring patterns and layouts [10–12] and mechanisms that improve the energy efficiency of neurons [13,14], circuits [15], and codes [16–19] have evolved in nervous systems, and these adaptations suggest that there is pressure on nervous systems to maximise performance and minimise expenditure on materials and metabolic energy [20].

Much of the metabolic energy consumed by a nervous system is used to generate and transmit signals, and most of this goes to the Na^+^/K^+^ pump, to restore the ionic concentration gradients that drive rapid electrical signalling and neurotransmitter uptake [21]. This energy usage is directly related to performance— more power is required to transmit signals at higher rates [22–24]. Furthermore, the quantities of energy used by neurons are sufficiently large to limit the coding, processing, and transmission of information. Thus the limited availability of energy not only constrains the size and total number of neurons in the brain [7,25], it limits representational capacity by placing a remarkably low ceiling on mean firing rates [21,26]. Although the balance between energy costs and performance could well play a formative role in the evolution of nervous systems, to our knowledge no single study has set out to establish these relationships by measuring both costs and performance across a set of comparable neurons.

Fly photoreceptors offer several advantages for such a systematic comparative study of the trade-offs between neuronal energy costs and neuronal performance. The biochemical and electrical signalling mechanisms, the phototransduction cascade [27], and the photoreceptor membrane [28,29], are exceptionally well described [30–32]. High quality intracellular recordings from identified photoreceptors in intact retina allow one to measure both cost and performance in the same cell. Performance can be measured directly, as the rate at which the photoreceptor transmits information, from recordings of voltage signals [33]. The metabolic cost of this information can be obtained by measuring membrane voltage and conductance, and then applying these measurements to a membrane model to calculate the ionic currents used to generate responses and the rate at which Na^+^/K^+^ pumps must consume ATP to maintain the ionic concentration gradients that drive electrical signalling. This empirical method yields the unit cost of information, measured in ATP molecules hydrolysed per bit of information coded [14,34].

We present a systematic comparative study of fly photoreceptors, which sets out to discover how neuronal energy costs change with neuronal performance. We compare homologous photoreceptors taken from four species of Diptera, the blowfly *Calliphora,* the fleshfly *Sarcophaga,* and two Drosophilids. The blowfly and the fleshfly have larger eyes with better spatial and temporal resolving power, presumably because these large flies fly faster and further and are more manoeuvrable than the Drosophilids. Photoreceptor performance is measured directly, as information throughput in bits s^−1^, and energy costs are estimated as the rate at which the Na^+^/K^+^ pump must hydrolyse ATP molecules in order to sustain signalling. We confirm that blowfly R1–6 photoreceptors achieve higher bit rates than D. melanogaster [31,33] at greater cost [14,34], and we extend this comparison to the full operating range of background light levels. Furthermore, by applying identical methods to four species, we describe how costs scale against performance. We find that it is costly to improve performance, because membrane conductance increases supralinearly with maximum bit rate, and this makes information more expensive in higher capacity cells. Our measurements confirm theoretical findings [16,18,35] that the fixed cost of maintaining a cell at rest, ready to signal, is a major determinant of metabolic efficiency and also establish a basic microeconomic relationship; namely that the fixed cost of maintaining a cell ready to signal increases with its maximum information rate. In this sense fly photoreceptors resemble cars; a high performance Porsche Carrera GT consumes three times as much fuel km^−1^ as a lower performance Honda Civic [36], even when driven at the same low speeds (urban cycle). Because this new example of a neuronal law of diminishing returns appears to be enforced by the basic biophysics of electrical signalling, we suggest that it operates in many neurons and could, therefore, play a significant role in determining the function, design, and evolution of nervous systems.

## Results

### Information Rates in Fly Photoreceptors

We compared information rates with energy costs in R1–6 photoreceptors from four species *C. vicina, S. carnaria, D. virilis,* and *D. melanogaster. C. vicina* and D. melanogaster R1–6 were chosen because they are known to transmit at very different rates. In daylight the large *Calliphora* cells transmit approximately 1,000 bits s^−1^ [30,33,34], whereas the smaller D. melanogaster cells transmit at just over 200 bits s^−1^ [31,32,37]. We developed a new preparation, the intact retina of *D. virilis,* to provide R1–6 cells of intermediate size and information rate. We also recorded from the large R1–6 photoreceptors of another vigorous fly with a large eye, S. carnaria, in order to confirm that the high costs measured in *Calliphora* R1–6 are associated with high information rates.

We measured information rates from intracellular recordings of voltage responses to optical signals (Figure 1) [33]. The photoreceptor was first adapted to a background light whose effective intensity had been calibrated as an effective photon rate by counting that same photoreceptor's discrete responses to single photons (see Materials and Methods; Figure 1A). This calibration takes account of differences in acceptance angle and sensitivity and enables us to compare the performance of photoreceptors receiving the same number of photons. Once stably adapted, the photoreceptor was presented with multiple repeats of the same brief sequence of pseudorandom modulation of the light around the background intensity (Figure 1B and 1C). The mean contrast of this modulation (standard deviation/mean) was 0.32, a value close to that of natural scenes (see Materials and Methods). Photoreceptors encode the fluctuations in stimulus contrast as a graded (analogue) modulation of membrane potential that is contaminated by noise. We extracted the photoreceptor's voltage signal (Figure 1B and 1C) by averaging the responses (averaging eliminates noise) and then extracted the noise by subtracting our estimate of the signal from the response to each stimulus repeat. Our estimate of the signal was transformed into the signal power spectrum *S*(*f*). Each of the extracted noise traces was transformed, and the resulting ensemble of spectra was averaged to generate the noise power spectrum *N*(*f*). Both signal and noise were distributed normally, allowing the rate at which the photoreceptor transmits information *I* in bits s^−1^ to be determined by applying Shannon's formula [38] to the power spectra of the signal *S*(*f*) and noise *N*(*f*):

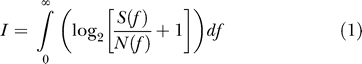
The logarithmic term in this equation is the distribution of information across frequencies, as plotted in Figure 1D.

**Figure 1:**
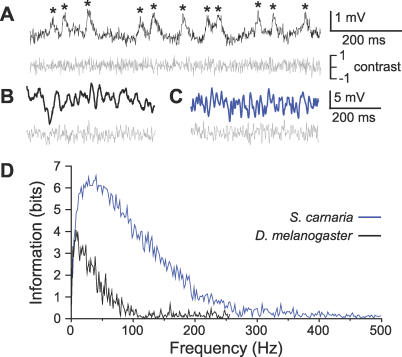
Intracellular Recordings of Voltage Responses and the Distribution of Information across Frequencies in R1–6 Photoreceptors of D. melanogaster and S. carnaria (A) Quantum bumps (*) recorded from D. melanogaster in response to continuous illumination by the white-noise stimulus (lower trace, grey), which was attenuated by 5.5 log units to give a mean effective photon rate of 9 s^−1^. (B) Average responses of a D. melanogaster R1–6 photoreceptor to 50 repetitions of a randomly modulated light of mean contrast 0.32. (C) The corresponding average response of an R1–6 photoreceptor from S. carnaria. Note that the responses in (B) and (C) have dissimilar waveforms because they were generated by different random sequences of intensity modulation, shown in grey beneath each voltage record. In both (B) and (C) the mean stimulus intensity was set to approximately 5 × 10^6^ effective photons s^−1^. Note that S. carnaria R1–6 responses (C) vary more rapidly than D. melanogaster (B). (D) This faster response gave the S. carnaria R1–6 a wider bandwidth, as demonstrated in (D) by plotting the distribution of information across response frequency for the two cells.

At the lowest photon rates (10^2^−10^3^ effective photons s^−1^) the information rates of all four photoreceptors were almost identical, suggesting that under these conditions the information rates in all four species were limited by photon noise, rather than response bandwidth (Figure 2). At higher photon rates (>10^3^ effective photons s^−1^) the information rates of the photoreceptors diverged (Figure 2). At the highest effective photon rates ∼10^7^ s^−1^, which are within 0.7 log units of the highest daylight intensities [39], *Calliphora* and *Sarcophaga* photoreceptors attained throughputs close to 1,000 bits s^−1^ (955 ± 70, *n* = 3 for *Calliphora* and 1,130 ± 67, *n* = 11 for *Sarcophaga*), compared with ∼510 bits s^−1^ in D. virilis photoreceptors (512 ± 26, *n* = 21) and ∼200 bits s^−1^ in D. melanogaster photoreceptors (197 ± 31, *n* = 26). Note that, as explained in the Materials and Methods, our set of values from 26 D. melanogaster photoreceptors includes data from 21 cells that were published in an earlier study [32]. The rates measured in *Calliphora* and D. melanogaster R1–6 photoreceptors are similar to those measured previously with comparable methods [31,34,37]. The information rate of D. melanogaster R1–6 photoreceptors saturated at our highest intensities, but the information rates in the other species did not (Figure 2). Nonetheless, because our highest photon rate is close to that experienced in full daylight [39], the photoreceptors are operating close to their natural intensity limit.

**Figure 2:**
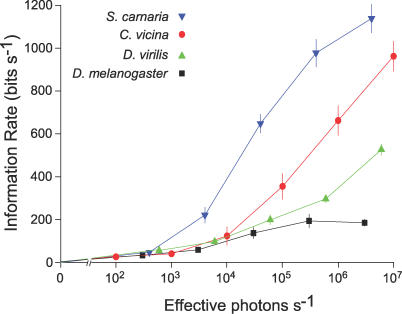
Comparison of Information Rates in R1–6 Photoreceptors from Four Dipteran Species Information rates (mean ± standard error of the mean) are measured from the response to a randomly modulated light of mean contrast 0.32, presented at five background (average) light levels to: S. carnaria (blue), C. vicina (red), D. virilis (green), and D. melanogaster (black). Each adapting light background was converted to effective photons s^−1^ to allow the photoreceptors to be compared under equivalent conditions.

The R1–6 photoreceptors of the larger more active flies, *Calliphora* and *Sarcophaga*, code information at higher rates because they maintain a higher signal-to-noise ratio (SNR) over a broader bandwidth of response. The contributions of SNR and bandwidth to performance are illustrated by comparing a plot of information versus frequency for the highest information rate photoreceptor, *Sarcophaga* R1–6, with a plot for the lowest information rate photoreceptor, D. melanogaster R1–6 (Figure 1D). At any given frequency the *Sarcophaga* R1–6 carries more information than the D. melanogaster R1–6 because its SNR, *S*(*f*)*/N*(*f*) in Equation 1, is larger. The *Sarcophaga* R1–6 codes almost half of its information at frequencies in the range 100–300 Hz but, because of its poorer bandwidth, D. melanogaster R1–6 codes very little information at frequencies above 100 Hz (Figure 1D).

### Metabolic Costs of Fly Photoreceptors

We used an established electrical model of the photoreceptor membrane to estimate the rate at which photoreceptors consume metabolic energy (see Materials and Methods). The model [14,34] incorporates the two major conductances, light-gated and potassium, as well as the electrogenic Na^+^/K^+^ pump, and calculates the flux of ions through these components from measurements of total conductance and membrane potential (Figure 3). The flux of ions through the Na^+^/K^+^ pump gives the rate at which ATP is hydrolysed in order to maintain ionic concentration gradients, and ATP hydrolysis rate in molecules s^−1^ is our measure of metabolic energy cost.

**Figure 3:**
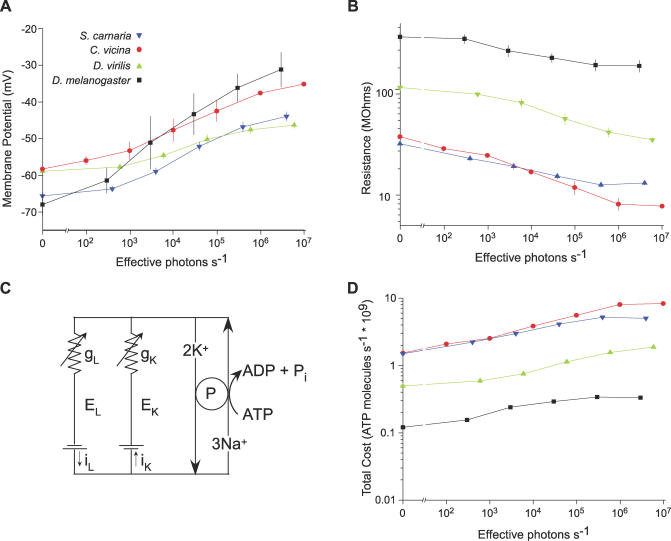
Measurements of Photoreceptor Membrane Properties Allow the Calculation of Metabolic Cost (A) The membrane potential (mean ± standard error of the mean) of R1–6 photoreceptors in the dark and at different effective photon rates, measured in four species S. carnaria (blue), C. vicina (red), D. virilis (green), and D. melanogaster (black). (B) The corresponding resistances (mean ± standard error of the mean) of R1–6 photoreceptor in the dark and at different effective photon rates. (C) The electrical model circuit of the photoreceptors. The model calculates from the measurements of membrane potential and resistance the rate at which the Na^+^/K^+^ pump, P, hydrolyses ATP molecules: *g*_L_ = light-gated conductance; *E*_L_ = reversal potential for light-gated current; *i*_L_ = light-gated current; *g*_K_ = potassium conductance; *E*_K_ = potassium reversal potential; *i*_K_ = potassium current. (D) The rate of hydrolysis of ATP molecules calculated at each effective photon rate for R1–6 photoreceptor of the four species (mean).

To obtain the data used to estimate metabolic costs, we measured a photoreceptor's membrane potential and input resistance (see Materials and Methods), first in the dark and then at each of the background light intensities at which we measured the information rate (Figure 3A and 3B). All photoreceptors were depolarised by light, and the steady-state depolarisation produced by a sustained background light increased with background intensity (Figure 3A). At lower backgrounds, below 10^3^ effective photons s^−1^, *Calliphora* R1–6 photoreceptors were most depolarised, and *Sarcophaga* R1–6 photoreceptors were least depolarised, whereas at higher backgrounds, above 10^5^ effective photons s^−1^, D. melanogaster R1–6 photoreceptors were most depolarised, and D. virilis R1–6 photoreceptors were least depolarised (Figure 3A). Photoreceptor input resistance dropped with increasing light intensity (Figure 3B) because of increased activation of light-gated channels and voltage-gated potassium channels [28,32]. In the dark, and at any particular photon rate, D. melanogaster R1–6 photoreceptors had the highest input resistance, D. virilis R1–6 were intermediate, and the *Calliphora* and *Sarcophaga* R1–6 photoreceptors had the lowest input resistances (Figure 3B).

Putting these measurements of membrane potential and resistance into our electrical model (Figure 3C), we obtained the rate of ATP consumption (Figure 3D). In the dark and at any particular photon rate, *Calliphora* and *Sarcophaga* R1–6 photoreceptors had the highest rate of ATP consumption, *D. virilis* R1–6 were intermediate, and D. melanogaster R1–6 photoreceptors had the lowest rate of ATP consumption (Figure 3D). Thus small photoreceptors that transmitted at lower bit rates (Figure 2) had lower energy costs (Figure 3D).

### Signalling Costs and Fixed Costs

We can separate photoreceptor energy cost into two components, dark and signalling. The dark cost is the rate at which ATP is hydrolysed to maintain the cell's resting potential in the dark. The signalling cost is the increase in ATP hydrolysis rate induced by light, i.e.,

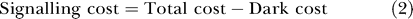



The signalling cost is, in microeconomic terms [40], a variable cost that increases with the level of output, whereas the dark cost is a fixed cost that, like the rent on a factory, is paid at a fixed rate, irrespective of output. In nervous systems the fixed cost of maintaining an inactive neuron's resting potential is an important determinant of the metabolic efficiency of distributed neural codes [16], of spike trains in single cells [18], and of stochastic signalling mechanisms such as ion channels and synapses [35].

The R1–6 photoreceptors of all four species have significant fixed costs—all photoreceptors consume substantial quantities of ATP in the dark (Figure 3D). This fixed cost differs greatly between species, according to photoreceptor input resistance and membrane potential. Comparing the photoreceptors of different species, although there are appreciable differences (up to 10 mV) in the dark resting potential (Figure 3A), the dark input resistances vary by more than an order of magnitude (Figure 3B) and are, therefore, primarily responsible for the large differences in ATP consumption rates in the dark (Figure 3D). The dark consumption is approximately 2 × 10^9^ ATP molecules s^−1^ in *Calliphora* and *Sarcophaga* R1–6 photoreceptors, whereas in D. melanogaster R1–6 the dark consumption is approximately twenty times less, 1 × 10^8^ ATP molecules s^−1^.

Illumination increases the rate of ATP consumption in all of the R1–6 photoreceptors and, as in the dark, those with the lowest input resistances (*Calliphora* and *Sarcophaga*) consumed the most ATP (Figure 3D). The similarity between the log–log plots of the total cost versus the effective photon rate suggests that the signalling costs of the photoreceptors from the different species have similar dependencies on light level and scale with the dark cost (Figure 3D). This suggestion led us to compare the dark costs and the signalling costs at different light levels, in the R1–6 photoreceptors of the four species.

In each of the four species, the photoreceptor signalling cost rises with intensity and approaches an asymptote at bright daylight levels (Figure 4A). Even though there was an approximately 25-fold difference in both the total and the signalling ATP consumption at the brightest light intensities across the four species, the ratio between the signalling cost at the brightest light levels (the maximum signalling cost) and the dark cost was similar in each photoreceptor and was, on average, 4.7 (Figure 4B). This scaling suggests that the energy consumption in the dark is directly related to the highest rates of consumption in bright light. When the signalling cost of a photoreceptor type is normalized with respect to dark consumption and plotted against the log of photon rate (Figure 4C), the curves for the different photoreceptor types are similar but not identical, as expected of a set of homologous photoreceptors that use similar mechanisms to generate and regulate responses, but fine tune these mechanisms to their particular requirements.

**Figure 4:**
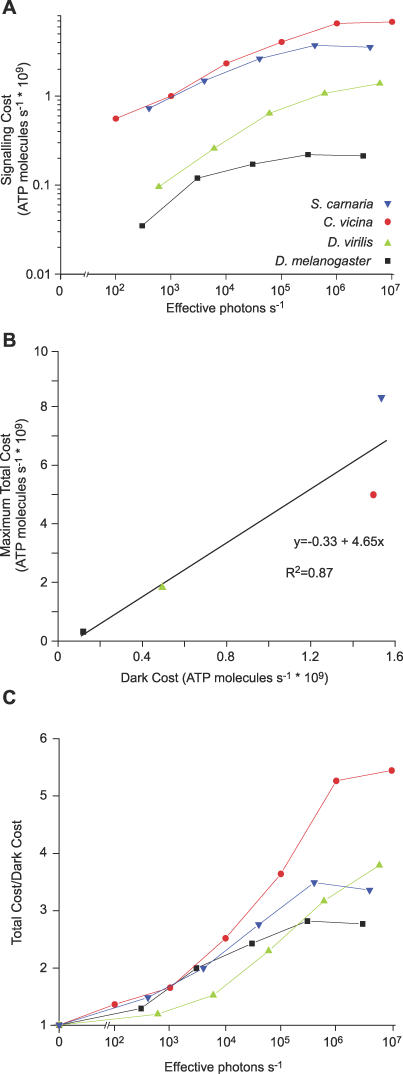
The Relationship between the Signalling Cost and the Fixed (Dark) Cost for R1–6 Photoreceptors from the Four Species *S. carnaria, C. vicina, D.virilis,* and D. melanogaster (A) The rate of hydrolysis of ATP molecules during signalling at each effective photon rate. (B) The maximum signalling cost versus the fixed cost for each of the four R1–6 photoreceptor types. The maximum is the signalling cost measured at the brightest light levels. (C) The ratio of total cost to fixed cost of each photoreceptor type at each effective photon rate.

### The Metabolic Cost of Information

By dividing the total rate of energy consumption (Figure 3D) by the corresponding rate of information transmission (Figure 2) at each light level, we derived the metabolic cost of information, as ATP molecules bit^−1^. This measure allows us to assess how economically each type of photoreceptor transmits information (Figure 5A). We discovered that all four photoreceptors showed the same behaviour: increasing the light intensity not only increases information rate (Figure 2), it also decreases the total cost per bit (Figure 5A). The proportional decrease is smallest in D. melanogaster R1–6, approximately 3:1, and largest in *Sarcophaga* R1–6 and *Calliphora* R1–6, approximately 10:1. A substantial part of this decrease in the total cost per bit can be attributed to the dark cost. At low light levels the dark cost is a substantial fraction of the total, and dividing this fixed cost by the low bit rate produces a high cost per bit, which then decreases as bit rate increases. At the highest light intensities the cost per bit starts to level out (Figure 5A), suggesting that under daylight conditions R1–6 photoreceptors are operating close to their minimum cost per bit.

**Figure 5:**
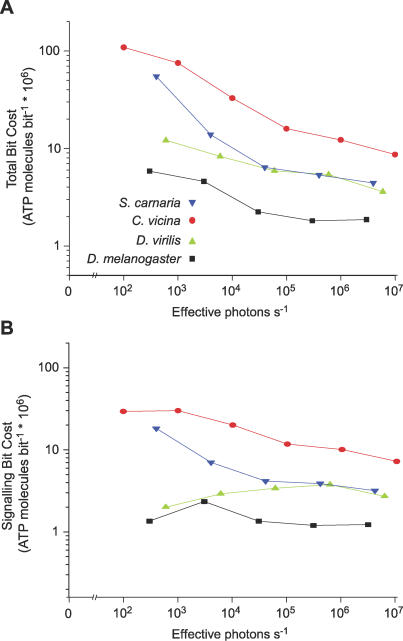
The Metabolic Cost of Information Decreases with Increasing Light Intensity (A) A double logarithmic plot of metabolic cost per bit at each effective photon rate and (B) a double logarithmic plot of the metabolic cost of signalling per bit at each effective photon rate are shown. Measurements are from R1–6 photoreceptors from four species, S. carnaria (blue), C. vicina (red), D. virilis (green), and D. melanogaster (black).

To see if other factors contribute to the fall in bit cost with increasing light level we calculated the signalling cost per bit, by dividing the rate at which ATP molecules are consumed for signalling by the bit rate. In the two high bit rate photoreceptors, *Sarcophaga* R1–6 and *Calliphora* R1–6, the signalling cost per bit is highest at low light intensities and then declines over the intensity range 10^2^–10^7^ effective photons s^−1^ to approximately 30% of its original value (Figure 5B). This increase in efficiency with photon rate is not observed in the two lower bit rate photoreceptors. In D. melanogaster R1–6 the signalling cost per bit first rises slightly with increasing intensity, peaks between 10^3^ and 10^4^ photons s^−1^ and then falls back to the previous level, while in D. virilis the signalling cost per bit doubles over the range 10^3^ to 10^6^ photons s^−1^ and then dips slightly (Figure 5B). Note, however, that over most of the intensity range the signalling cost per bit is lower in the low bit rate cells (Figure 5B). The signalling cost per bit could be falling with increasing light level in the *Calliphora* and *Sarcophaga* R1–6, because these photoreceptors expand their bandwidth to higher frequencies (e.g., Figure 1D) to achieve higher bit rates. Contributions to a fall in cost per bit could also be made by the improvement in photoreceptor SNR and by light adaptation of the phototransduction cascade, which, by reducing the light-gated conductance activated per photon [41], reduces the energy cost per photon.

### Cost and Performance

By plotting bit cost versus bit rate (Figure 6) we are able to compare the efficiency of cells operating at the same information rate. The total cost per bit varies consistently between the different species. At a given information rate D. melanogaster R1–6 photoreceptors encode most economically, it is approximately three times more expensive to operate D. virilis R1–6 photoreceptors at the same information rate, and approximately ten times more expensive for *Calliphora* and *Sarcophaga* R1–6 photoreceptors. These proportional differences in cost are, to a first approximation, maintained over the range of bit rates, and this suggests that the higher total cost per bit in the two larger flies, *Calliphora* and *Sarcophaga* (Figure 6A)*,* is primarily associated with their higher dark cost. Because signalling cost tends to increase with dark cost (Figure 4), the signalling costs per bit are also substantially higher in *Calliphora* and *Sarcophaga* R1–6 than in Drosophilid photoreceptors operating at the same bit rate (Figure 6B).

**Figure 6:**
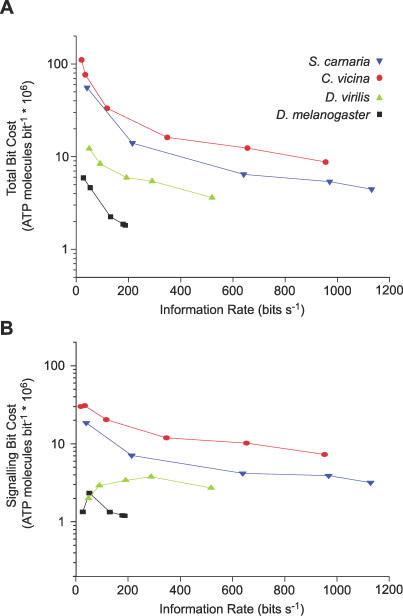
The Metabolic Cost of Information in R1–6 Photoreceptors Decreases When the Information Rate Is Increased by Raising the Light Level (A) The metabolic cost per bit plotted logarithmically versus the bit rate for R1–6 photoreceptors of the four species S. carnaria (blue), C. vicina (red), D. virilis (green), and D. melanogaster (black). (B) The metabolic cost of signalling per bit plotted logarithmically versus the bit rate for R1–6 photoreceptors in the four species.

The information rates measured with our brightest stimuli are indicative of a photoreceptor's maximum performance. Comparing metabolic costs with these highest rates, we see that both the total cost and the dark cost increase supralinearly with performance. Plots of the logarithms of costs against the logarithms of highest rates (Figure 7) suggest that the total cost increases as (performance)^1.7^, and the dark cost increases close to (performance)^1.5^, but with only four species these exponents are preliminary estimates. Nonetheless, there is no doubt that both the unit cost of information (the total cost per bit) and the dark cost are directly related to a photoreceptor's ability to transmit information (Figures 5 and 6); the higher a photoreceptor's maximum bit rate, the higher the dark cost, the higher the signalling cost, and the higher the total cost per bit.

**Figure 7:**
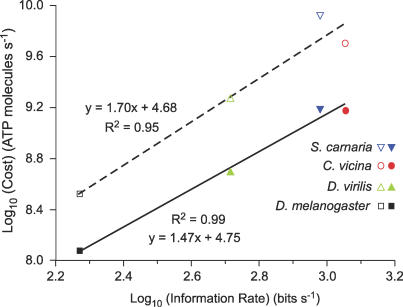
The Scaling of Metabolic Cost with Performance in Dipteran R1–6 Photoreceptors The logarithms of the total cost (open symbols) and the fixed cost (solid symbols) are plotted against the logarithm of maximum information rate. Costs are in ATP molecules hydrolysed per photoreceptor per second. Each data point represents the mean values from R1–6 photoreceptors in one of the four dipteran species used in this study. The linear fits suggest that the total cost of photoreceptor signalling (dashed line) increases as (information rate)^1.7^, and the fixed cost of maintaining the photoreceptor in the dark (solid line) increases as (information rate)^1.47^.

In conclusion, our recordings from intact fly photoreceptors demonstrate that the cost per bit varies with bit rate in two ways. In any single photoreceptor (e.g., a *Calliphora* R1–6), bit rate increases with light level while the total cost per bit falls (Figure 5). This fall is due to two factors, offsetting the dark cost (the substantial fixed cost of maintaining the photoreceptor's resting potential in darkness) and in the high bit rate photoreceptors, a substantial reduction in signalling cost per bit (Figure 5). However, when we compare homologous R1–6 photoreceptors in different species, we see that the energy cost per bit increases with a photoreceptor's performance (Figure 6), where performance is assessed from the highest information rate measured with our brightest stimulus. Again, the relatively high fixed cost of the dark resting potential (Figures 3, 4, and 7) is implicated in this relationship between maximum bit rate and bit cost.

## Discussion

We have investigated relationships between information rate and energy expenditure in single neurons by comparing homologous R1–6 photoreceptors from four species of fly. The highest throughput of information varied according to species, from ∼200 bits s^−1^ in D. melanogaster R1–6 photoreceptors to ∼1,000 bits s^−1^ in *Sarcophaga* and *Calliphora* R1–6 photoreceptors. This 5-fold increase in performance is accompanied by a 25-fold increase in energy consumption, an order of magnitude increase in the energy cost per bit and a similar increase in the cost of maintaining the photoreceptor's resting potential in the dark. This dark consumption constitutes a fixed cost because it is paid continuously, irrespective of the rate at which the photoreceptor is transmitting information. These results demonstrate that information is more expensive in high performance neurons, because both the fixed cost and the signalling cost increase supralinearly with signalling ability. Thus efficiency declines with increasing capacity, as found in two other physiological systems, mitochondria [42] and muscle fibres [43].

We will discuss the validity of the experimental methods that we applied to photoreceptors in order to establish that cost increases with capacity. We then turn to the biophysical basis of the relationship between energy cost and performance and its possible effects on photoreceptor structure and function. We will conclude by considering how our measurements of energy-information trade-offs might advance our understanding of the design and evolution of nervous systems.

### Relevance and Reliability of Our Measures

It is important that our measures of information rate and metabolic cost are biologically relevant and reliable. We argue that information rate, in bits s^−1^, is both a convenient and an appropriate measure of photoreceptor performance, despite the fact that Shannon's treatment of information rate (Equation 1) treats all parts of the signal equivalently, irrespective of the features they represent. Most of the features extracted by fly visual systems are unknown to us, nonetheless there are three reasons why we are confident that the “feature-free” measure, information rate in bits per second, is appropriate. First, unlike the R1–6 photoreceptors of the male housefly lovespot, which are specialised to detect rapidly moving high contrast targets [44], the R1–6 photoreceptors in our species do not appear to be adapted to detect particular features. Second, R1–6 photoreceptors support many aspects of vision because they feed a variety of parallel circuits in the optic lobes [45]. Third, even when visual systems are devoted to processing a small number of biologically relevant objects, photoreceptors still have to code a wide range of signals. The range is wide because a photoreceptor signals the presence of an object in its field of view as a change in photon rate. This change varies greatly in relative amplitude (contrast) and time course, depending on the object's position, illumination, orientation, distance, movement, and the background against which the object is viewed. Thus, because viewing conditions cause a single object to generate a range of photoreceptor signals, the general measure of performance, bit rate, is appropriate. The argument for this general measure is strengthened still further by the fact that synaptic transfer from photoreceptors to interneurons is optimised to maximize bit rate [46–48]. Finally, bit rate takes account of the basic biophysics of coding by combining two more fundamental determinants of signal quality: the accuracy of response and the ability to follow rapidly changing signals. Accuracy contributes to information rate through the SNR, and response speed contributes by determining the bandwidth over which signals can be transmitted (Equation 1).

Given that information rate, in bits per second, is an appropriate measure of photoreceptor performance, are the highest bit rates reached in bright light (Figure 2) adequately representing the different abilities of the four photoreceptors to code information? Our experiments were designed to apply the two determinants of information rate, bandwidth and SNR, equally to all photoreceptors. White noise tests the full bandwidth by injecting equal power at frequencies that extend well beyond each cell's cutoff. By using photon counts to compare photoreceptors from different species, we ensured that our comparisons are not biased by optical differences (e.g., in facet lens diameter, focal length, and rhabdomere width) that influence the number of photons individual photoreceptors receive from the same stimulus [49,50] and hence the photon noise limit to SNR.

Although our stimuli enable us to compare photoreceptors on equal terms, there are two reasons why our measured information rates fall short of full capacity. With the exception of D. melanogaster, we were unable to saturate photoreceptors' information rates (Figure 2), even though our highest intensities are within a factor of five of the photon rates experienced in full daylight [39]. In addition, the information capacity is, by definition, determined using a stimulus that is tailored to distribute power optimally across the photoreceptor bandwidth. Our bit rates in *Calliphora* (Figure 2) are 50% below the capacities measured at the same photon rates [33] but, although our white-noise stimuli underestimate information capacity, they overestimate the rates generated under natural conditions because natural stimuli have less power at high frequencies. However, the overestimate appears to be small (10%–20%) [51] compared with the 5-fold differences in the highest rates measured in the photoreceptors of the four species (Figure 2). We conclude that, although the highest information rates we measured underestimate full capacities, our data reflect the highest rates expected under natural conditions. We can, therefore, conclude that our measured bit rates adequately describe differences in photoreceptor performance between species.

Turning to our comparison of metabolic cost, the measurements of input resistance and membrane potential used to calculate energy consumption were consistent from cell to cell and agreed with previous studies of *Calliphora* and D. melanogaster [28,30,32,52]. As expected, the larger photoreceptors with lower input resistance consume more energy. Our conductance-based method underestimates total energy consumption, because it neglects both the intermediate processes in the phototransduction cascade that consume energy [27] and essential maintenance processes, such as macromolecular synthesis. However, the intermediate processes of phototransduction are likely to add less than 10% to the total energy cost because ion flux is the final stage in signal amplification [34] and, in active neural tissue, macromolecular synthesis contributes less than 10% to the total energy consumption [21]. Most importantly, our estimate of energy consumption for a fully light-adapted *Calliphora* photoreceptor, 7 × 10^9^ ATP molecules s^−1^, agrees remarkably well with the most recent value obtained from measurements of retinal oxygen consumption, 6.5 × 10^9^ ATP molecules s^−1^ [53].

### The Biophysical Basis of Trade-Offs between Energy and Information

A number of studies suggest that energy cost and performance are related to photoreceptor structure and biophysics via two fundamental measures of signal quality, SNR and bandwidth. These two measures determine the measure of performance adopted in this study, information rate (Equation 1), and the photoreceptors that achieve higher rates do so because they have a better SNR in bright light and a wider bandwidth (Figure 1D). Photoreceptor SNR rises with the rate at which photons are being transduced, and in insect photoreceptors the SNR often tends to plateau at the highest light levels, as photomechanical mechanisms attenuate the incoming photon flux to prevent saturation. The proposal that the maximum attainable SNR has a structural basis, the number of photoreceptive microvilli in a photoreceptor [54], is strongly supported by more recent evidence. A microvillus contains all of the signalling molecules of the phototransduction cascade, and a single photon hit appears to produce an all or nothing response, a quantum bump, from one entire microvillus [27,41]. The corollary that the maximum rate of photon conversion, and hence the maximum SNR, is limited by the number of microvilli, is supported by measurements of SNR under saturated conditions [39] and by the observation that photoreceptors with more microvilli achieve higher SNRs [31,32,55].

The second determinant of information rate, bandwidth, is regulated by two sets of factors, the molecular dynamics of phototransduction and the electrical properties of the photoreceptor membrane [56–58]. Photoreceptors regulate their bandwidth by controlling the dynamics of the phototransduction cascade and by tuning the frequency response of the membrane with voltage-gated potassium channels [28,31,32,59]. In a given photoreceptor, the bandwidth is adjusted according to light level to improve information throughput, and, comparing different photoreceptors, the maximum bandwidth varies systematically according to retinal position, colour type, and visual ecology [30,55,60]. Thus photoreceptor bandwidth is carefully regulated to adapt response dynamics to operating conditions.

These two factors, the number of microvilli and the membrane bandwidth, link information rate to energy consumption. Increasing the number of microvilli to improve the SNR will increase the photoreceptor's membrane area and hence its total conductance and capacitance, leading to larger ionic currents. Increasing the membrane's potassium conductance to widen its bandwidth (by reducing its time constant) also increases the flow of ions across the membrane. Indeed, the high metabolic cost of increasing membrane bandwidth has been invoked to explain why slowly flying insects, exemplified by Tipulid flies, have slow photoreceptors with a low potassium conductance, long time constant, and narrow bandwidth [59,60]. The low potassium conductance of slow cells is achieved by inactivation [58], and the contribution of potassium channel inactivation to energy efficiency has been demonstrated directly by genetically manipulating and modelling photoreceptors in *D. melanogaster.* When the rapidly inactivating *Shaker* K^+^ -channel of R1–6 photoreceptors is deleted by mutation, there is an increase in tonic conductance, and the cost of information, in ATP molecules bit^−1^, increases [14]. These findings strongly suggest that the biophysics of SNR and bandwidth link information rate to energy consumption to produce the trade-off between cost and performance observed here. However, this suggestion must be confirmed by relating measurements of SNR, microvillus number, membrane conductance, and membrane bandwidth to measurements of cost and capacity. Such a detailed analysis of photoreceptor structure, biophysics, and performance will reveal whether large increases in bandwidth explain the fall in signalling cost per bit seen in high bit rate photoreceptors (Figures 5B and 6B). This detailed comparison will also decide whether fly photoreceptors divide their energy investment between SNR and bandwidth optimally, to maximize energy efficiency.

### The Significance of Fixed Costs

To the best of our knowledge, this is the first study to measure the fixed cost of maintaining a neuron ready to signal and then relate this fixed cost to performance and metabolic efficiency. The fixed cost of maintaining a photoreceptor's dark-resting potential is high (approximately 20% of the cost in full-daylight) and, following earlier calculations [39], we find that in *Calliphora* this dark current amounts to approximately 2% of a blowfly's total resting metabolic rate. Although photoreceptor fixed costs vary between species by more than an order of magnitude, they are a remarkably constant proportion, between a fifth and a quarter, of the energy consumed in full daylight (Figure 4). This proportion suggests that the metabolic scope of insect photoreceptors (the ratio between the maximum sustainable metabolic rate and the resting metabolic rate) lies between four and five. Fixed costs increase with capacity (Figure 7), as also observed in comparative studies of energy throughput and metabolic rate in mice [61]. Species of mice that are adapted to live in areas where food is more plentiful convert food to energy at higher rates than species that live in areas where food is scarce. The high-energy users also have higher basal metabolic rates, presumably to support the extra fixed cost of the larger organs, such as gut and heart, needed to handle higher rates of energy throughput [61].

The reasons why R1–6 photoreceptors have a high fixed cost are unclear, but the proximate cause is a dark resting potential that is approximately 20 mV less negative than the potassium reversal potential [28]. The inward currents that produce this depolarisation have not been identified, but the limited evidence suggests two possibilities, both of which are related to maintaining a high sensitivity. The first source is spontaneous activation of the phototransduction machinery [62,63]. Because this spontaneous activity increases with the number of microvilli, its cost will increase with capacity. The second source of inward current could be voltage-sensitive conductances and feedback synapses associated with signal amplification and band pass filtering at the photoreceptor's high sensitivity output synapses [64,65], which are tonically active in the dark [66]. We note in passing that strictly diurnal insects could economise on energy consumption by down-regulating the phototransduction cascade and reducing synaptic activity at night. The fact that a photoreceptor's fixed costs could be related to both its input and its output emphasises that energy efficiency is a systems' property that depends upon relationships within and between components [15,35,67].

### Energy, Information, and the Evolutionary Adaptation of Insect Retina

The energy-information trade-offs that we have described in photoreceptors have implications for the design and evolution of insect retinas. The cost of increasing the maximum rate at which a photoreceptor can handle information is substantial and involves large increases in both the cost per bit and the fixed cost of maintaining the photoreceptor in the dark. This leads to a law of diminishing returns whereby a small increase in information capacity requires a larger proportional increase in energy cost. This law increases evolutionary pressure to reduce photoreceptor performance to the minimum required for satisfactory visually-guided behaviour by penalizing excess capacity. The result, allocation of resources according to need, could help to explain why, in males of *Calliphora vicina,* the R1–6 photoreceptors that look ahead at approaching objects through superior optics have higher information rates than those looking sideways and backwards through inferior optics [30].

The fixed costs of phototransduction could be particularly important for nocturnal insects. Their photoreceptors often have a large area of photosensitive membrane to improve photon capture [49,68,69], and this could create problems due to high fixed costs. Furthermore, nocturnal photoreceptors operate at extremely low light levels where fixed costs make each bit of information extremely costly (Figures 4 and 5). Because the membrane area of the photoreceptive microvilli cannot be sacrificed without losing photons, the only way to reduce fixed costs is to reduce membrane conductance. In extreme circumstances this could result in the photoreceptor membrane having such a long time constant that this, rather than the number of microvilli, limits information capacity at higher light levels. The photoreceptors of nocturnal Tipulids have high resistances and long time constants [58,60] and may well, therefore, be implementing this strategy.

### Energy, Information, and the Design and Evolution of Nervous Systems

The relationships between energy and information observed here in fly photoreceptors will apply to signalling systems that share similar biophysical relationships between SNR, bandwidth, and energy cost. Although neurons use synapses as discrete signalling units, rather than microvilli, they too are subject to the stochastic activation of conductance, and are constrained by membrane time constant [70]. Consequently, improvements in neuronal reliability, speed of response, and information rate will probably involve increased energy consumption; namely the additional signalling cost of operating extra channels and synapses and the additional fixed cost of this extra signalling machinery. Recent experiments on spiking neurons support the suggestion that additional signalling and fixed costs make information more expensive in neurons that transmit at higher rates [71]. Comparing the different classes of ganglion cell in guinea pig retina, brisk cells transmit information at higher rates than sluggish cells, because brisk cells fire spikes more frequently with greater temporal precision. Information will be more expensive in a brisk cell because, as expected of a cell that fires at a higher rate, a spike in a brisk cell carries less information than a spike in a sluggish cell [71]. In addition, because brisk cells are larger than sluggish cells, a brisk cell spike will use more energy. By analogy with fly photoreceptors, we further suggest that fixed costs will be higher in brisk cells, because their superior temporal precision requires more channels and synapses leading to a higher baseline conductance. This extra conductance will also increase the signalling cost of generating spikes. For these reasons information will cost more in the higher rate brisk cells than in the lower rate sluggish cells. This cost differential could help to explain why the retinal output is divided among different classes of ganglion cell with over 60% of the information being transmitted by low cost sluggish cells [71]. Thus, this classic example of parallel coding could be improving energy efficiency by directing the signals that require less temporal precision into lower cost channels [72,73]. This design principle could well extend beyond the retina to higher visual centres and to the coding of other sensory modalities, such as hearing.

The relationships between fixed costs, signalling costs, and bit rate could have a significant impact on coding and neural circuit design. In fly R1–6 photoreceptors, the fixed and total costs increase as power functions of maximum bit rate, with exponents of approximately 1.5 and 1.7, respectively (Figure 7). Thus the relationship between cost and performance follows the law of diminishing returns. Similar examples of this law have been observed in theoretical studies of spiking neurons, synaptic arrays, and neural circuits [15,16,34]. In general [18], this law makes it advantageous to implement energy efficient neural codes [17] that distribute information among spikes or neurons so as to avoid high rates. The relationship between costs and capacity measured in fly photoreceptors demonstrate that this law applies not only to signalling costs, but also to fixed costs. Again, theoretical studies have shown that fixed costs are important determinants of energy efficiency, which help set the optimum numbers of synapses and channels [35] and the optimum sparseness of energy-efficient neural codes [16]. Thus the empirical data presented in this study (Figure 7) demonstrate a relationship between representational capacity (i.e., highest bit rate) and fixed cost, which will influence the energy efficiency of circuits and codes.

Information rate is not the only measure of neuronal performance by which to judge efficiency. The measures that are most appropriate for a neuron will be defined by the role the neuron plays, processing signals in circuits, and determining behaviour. Relevant measures of performance could include the sharpness of frequency tuning in auditory systems [74], latency in reflex arcs [75], and storage capacity in cortical networks [76]. Just as the basic biophysical constraints of bandwidth and noise link photoreceptor information rate to energy consumption, so might improvements in these other performance measures involve additional costs. As examples, frequency tuning could be linked to ion flux by the conductances used to regulate the membrane time constant and to actively suppress or amplify particular frequency bands, while rapid responses and high temporal precision are associated with shorter time constants, larger diameter cells, and larger synapses [77]. On this basis, we suggest that comparative studies of neuronal cost and performance, similar to the experiments presented here, will confirm that trade-offs between energy cost and performance are widespread.

The balance between energy cost and performance appears to have played a significant role in determining the evolution of nervous systems [5,6]. Numerous examples exist of the relative reduction or expansion of the whole brain or particular brain regions during evolution [7,78]. For example, in the extinct bovid genus *Myotragus,* brain size was reduced by 50% relative to similar bovids of comparable body mass following isolation on a Mediterranean island. It is argued that this reduction was a response to two factors; reduced predation pressure and increased competition for a limited food supply [79]. In birds the degree of specialization for food hoarding correlates with the volume of the hippocampus, expressed relative to both body mass and telencephelon volume [80]. Thus both energetic costs and behavioural requirements are likely to be important selective pressures influencing relative brain size [7]. Improvements in behavioural performance can come about in at least three ways; by acquiring more information from the environment, by improving the nervous system's ability to process and represent information, and by finer or more appropriately coordinated control of motor outputs and muscles. The energy-information trade-offs discovered in fly photoreceptors R1–6 demonstrate that even small improvements in the ability of single cells to acquire and transmit information, and hence to process information more accurately and rapidly, come at a high energetic cost. Costs rise more rapidly than performance and this intensifies selection on neural structures and promotes evolutionary adaptation by increasing the sensitivity of trade-offs between costs and benefits.

## Materials and Methods

### Animals and preparation.

We used four species of fly for this study; *C. vicina, S. carnaria, D. virilis,* and D. melanogaster. Populations of three of these species *C. vicina, D. virilis*, and D. melanogaster were maintained in the Department of Zoology, University of Cambridge, United Kingdom. Individuals of S. carnaria were obtained from wild populations near Cambridge between May and September, 2004. The two larger fly species, C. vicina and *S. carnaria,* were mounted with their dorsal surface uppermost on a wax platform. Additional wax was used to fix the head and thorax but not the abdomen, which was left free to allow breathing. Both *Drosophila* species were mounted in a custom-built holder, and their head and thorax fixed using wax. In all species a small window (no more than a few facets in diameter) was cut manually into the top of the right compound eye and sealed immediately with silicon grease to prevent dehydration. The grease is soft enough to allow intracellular microelectrodes to be inserted through the seal, without damage. A second window was cut into the left compound eye to allow access for the indifferent electrode, a 50-μm-diameter silver wire.

### Intracellular recordings.

In vivo intracellular microelectrode recordings were obtained from R1–6 photoreceptors of *C. vicina, S. carnaria, D. virilis,* and D. melanogaster. All recordings were made using borosilicate glass electrodes filled with 3 M KCl. The electrode resistance varied considerably depending on the species from which the recording was being made; electrodes with resistances of 100–130 Ω were used for C. vicina and S. carnaria R1–6 photoreceptors, whereas 200 Ω or greater resistance electrodes were used for R1–6 photoreceptor recordings from D. virilis and D. melanogaster. The pipettes were pulled from 10-cm borosilicate glass capillaries (1.0 mm outer diameter, 0.58 mm inner diameter; GC100F-10, Harvard Apparatus, http://www.harvardapparatus.co.uk) using a Sutter P97 puller (Sutter Instruments, http://www.sutter.com) and inserted into the eye, through the silicon grease seal using a Zeiss Jena grease-plate micromanipulator. All recordings were made using an Axoclamp 2A amplifier (Molecular Devices, http://www.moleculardevices.com). Throughout recordings the temperature of the flies was maintained between 22 °C and 24 °C.

Photoreceptors were considered for analysis only if their membrane potentials were hyperpolarised by more than −55 mV in the case of photoreceptors from the drosophilid species and −60 mV in the case of photoreceptors from C. vicina and S. carnaria. Additional criteria such as the amplitude of the saturating impulse response in dark-adapted conditions and the photoreceptor input resistance were also used to determine recording quality. The photoreceptor responses to light were recorded in bridge mode. To determine the input resistance, current was injected, and the voltage response measured in switched current clamp mode. Stimulus generation and data acquisition were carried out using a digital computer and a purpose-built interface. Both stimuli and responses were usually digitised at 2 kHz and, to prevent aliasing, responses were low pass filtered by a four-pole Butterworth with a cutoff at half the Nyquist frequency, i.e., 500 Hz.

### Optical stimulation.

Photoreceptors were stimulated by a point source, the tip of a light guide that was positioned on the optical axis and subtended six degrees at the cornea. In the setup used for *Calliphora* R1–6, white light was provided by a 450-W high-pressure xenon arc lamp (PRA model 301s), which was stabilised with optical feedback to suppress unwanted fluctuations in the light intensity delivered to the waveguide to below 0.5% (root mean square). To provide white-noise stimulation, the arc was modulated by feeding a voltage command waveform from the computer to the optical feedback unit. In the setup used for the other photoreceptors, the light source was a high intensity LED (505 nm, LEDtronics, http://www.ledtronics.com) whose output was controlled directly by a voltage to current converter, driven directly by the computer. All voltage commands were corrected for the nonlinear characteristics of the LED. Light was attenuated by calibrated neutral density filters to provide a series of background light levels.

### Calibrating photoreceptors by counting quantum bumps.

The effective intensity of the light source was determined for each photoreceptor by counting its responses to single photons, quantum bumps [81]. The photoreceptor was dark-adapted for at least 20 min, until it was sufficiently sensitive to produce clearly resolvable bumps (Figure 1A). The light level was then adjusted to produce from three to ten quantum bumps per second, by inserting neutral density filters, and the bump rate determined by counting at least 100 bumps in a measured time interval. The background light level was increased in steps by removing neutral density filters, and the effective photon rate at each background was extrapolated by multiplying the bump rate by the reduction in filter attenuation.

### Measuring information rates.

Information rates were measured from a photoreceptor's voltage response to Gaussian white noise [33] using well-established procedures [30–32,34]. The light source was modulated randomly for 0.512 s with a contrast *c*(*t*) *= I*(*t*)*/I*_o_, where *I*(*t*) and *c*(*t*) specify the intensity and contrast with time *t,* and *I*_o_ is the mean light level. The root mean square contrast was 0.32, which is close to the mean value of 0.4 measured for natural scenes [46]. The voltage waveform used to modulate the light source was generated digitally, by inverse Fourier transformation of a spectrum with constant amplitude and random phase, up to a cutoff frequency of 500 Hz. To iron out small inconsistencies in signal power spectra, three pseudorandom Gaussian time traces, *I*(*t*) were used, each repeated 50 times. This was reduced to two pseudorandom traces in *D. melanogaster,* where controls showed that this reduction had a negligible effect on measured information rates. The ensemble average of the photoreceptor voltage response to each sequence was derived to give the voltage signal *S*(*t*) (Figure 1B and 1C), and this estimate of signal was subtracted from each of the responses to derive 50 noise traces. The noise traces were transformed to power spectra and ensemble averaged to give the noise power spectrum, *N*(*f*), which was corrected for recording noise by subtracting the noise spectrum recorded with the electrode outside the cell. The two or three signal traces were transformed, and the spectra averaged to give the signal power spectrum, *S*(*f*)*.* A four-term Blackmann-Harris window was applied to the signal traces and the noise traces prior to transformation to the frequency domain, and the SNRs, *S*(*f*)*/N*(*f*), were corrected for statistical bias [82]. The amplitude distributions of signal and noise were approximately Gaussian and could, therefore, be used to calculate the information rate according to Equation 1. To improve the reliability of our conclusions, the measurements of information rates made in the five D. melanogaster photoreceptors R1–6 recorded for this study were supplemented with published data from 21 cells [32], giving 26 cells in all. The information rates obtained from the five new cells were very similar to those obtained earlier, despite having been obtained on a different setup.

### Measuring membrane resistance.

The membrane resistance was measured from recordings of the photoreceptor membrane's voltage response to current that was injected via the recording electrode, using a discontinuous switched clamp. In the experiments performed on *Calliphora* the resistance was estimated from the response to injected white-noise current, because this method also measures the dynamic impedance of the membrane [83]. The current was modulated using digitally generated waveforms, as described above for the white-noise optical stimulus. The pseudorandom current sequences had a zero mean, and their root mean square amplitude was adjusted to recording conditions, to generate a peak-to-peak membrane response of 2–4 mV. The average voltage response to current *v*(*t*) was calculated by ensemble averaging 200 repeats of the pseudorandom white-noise stimulus and transformed to the response power spectrum *V*(*f*). Dividing *V*(*f*) by the power spectrum of applied current *i*(*f*), yielded the impedance *Z*(*f*). The membrane resistance, *R*_M_, was estimated from the zero frequency asymptote of *Z*(*f*)*.* In the other three species, the photoreceptor membrane resistance was measured from the change in membrane potential produced by a low amplitude current pulse. The pulse's duration was adjusted so that it fully charged the membrane capacitance and, to ensure that the activation of voltage-sensitive conductance had a negligible effect on these measurements, the current was reduced to a level (∼50 μA), where positive and negative pulses produced symmetrical responses. The responses to several hundred current pulses were averaged to generate a reliable estimate of the voltage change. By using very small currents, this second method returns a value of membrane resistance that is closer to the steady state because it reduces artefacts due to rectification. This may explain why the resistances measured in *Sarcophaga* R1–6 using current pulses are slightly higher than those measured in *Calliphora,* using white-noise current (Figure 3), even though *Sarcophaga* achieves high information rates (Figure 2). In addition the smaller currents have less of a deleterious effect on recording stability.

### Calculating photoreceptor ATP consumption.

ATP consumption was estimated by applying measurements of membrane resistance and potential to a standard membrane model (Figure 3) of the insect photoreceptor [34]. Additional data on the membrane resistance and potential of D. melanogaster photoreceptors were obtained from the literature as follows. The single set of values of membrane resistance versus background intensity reported by Juusola and Hardie [31] were added to the measurements from five cells obtained for the present study, to give sets of values for six cells. We took 21 sets of measurements of membrane potential versus background light intensity from an earlier study by Niven et al. [32], which, with the five new cells recorded for this study, gave 26 sets of values in all.

The model incorporates the three dominant membrane mechanisms, a light-gated conductance *g*_L_ with reversal potential *E*_L_ = −5 mV [84], a potassium conductance *g*_K_ with reversal potential *E*_K_ = −85 mV [28], and a standard Na^+^/K^+^ pump that generates a pump current, *i*_p_ by exporting three Na^+^ ions and importing two K^+^ ions per ATP molecule hydrolysed [85,86]. When the photoreceptor is in the steady state and has a membrane potential *E*_M_, the light-gated conductance and the potassium conductance produce transmembrane currents *i*_L_ = (*E*_m_ − *E*_L_)*g*_L_ and *i*_K_ = (*E*_m_ − *E*_K_)*g*_K_. In order to maintain ionic homeostasis the pump current must be *i*_p_ = *i*_K_/2.

The pump current can be derived from the membrane model by equating currents across the model membrane *i*_K_ + *i*_L_ + *i*_p_ = 0 setting *g*_K_ + *g*_L_ = 1/*R*_M_ where *R*_M_ is the measured membrane resistance, and inserting the measured membrane potential, *E*_M_. The rate of ATP hydrolysis required to generate this steady-state pump current is our estimate of the rate at which the photoreceptor consumes energy.

### Measurement protocols.

Following a stable electrode penetration, the photoreceptor was dark adapted for at least 20 minutes and then calibrated by counting quantum bumps (Figure 1A), as described above. A neutral density filter was removed from the light beam to set the first background light level, the background light was switched on, and the cell was adapted for at least 2 min, until its membrane potential reached a stable steady state. Once the photoreceptor was stably light adapted, the membrane potential *E*_M_, the impedance *Z*(*f*), and the information rate were successively measured, using the procedures described above. The membrane potential was then checked for drift. The light was then extinguished, the stability of the resting potential was checked for 30 s–1 min, and following withdrawal of another neutral density filter, the next highest background was switched on. This sequence of stable light adaptation and measurement was repeated until the maximum effective intensity was reached. The light was then extinguished, and the cell left in the dark to check that the resting potential returned to a value that was within 2 mV of that measured at the start of the experiment. Data from cells that failed this final test were rejected. Finally, the electrode noise was measured with the electrode just outside the cell, in a position where the noise amplitude was at a minimum.

## Abbreviations

SNR: signal-to-noise ratio
